# Supporting unpaid carers around hospital leave for people detained under the Mental Health Act (1983) in England: carer and practitioner perspectives

**DOI:** 10.1186/s12888-024-05602-9

**Published:** 2024-02-23

**Authors:** Nicola Moran, Ruth Naughton-Doe, Mark Wilberforce, Emma Wakeman, Martin Webber

**Affiliations:** 1 School for Business and Society, University of York, YO10 5DD York, England; 2 St Andrew’s Healthcare, Billing Road, NN1 5DG Northampton, England

**Keywords:** Hospital leave, Mental Health Act, s.17, Unpaid carers, Inpatient, Detention, Practitioners, S.17 Standard, Coercion

## Abstract

**Background:**

When an individual is detained in hospital it is important that they maintain contact with their family, friends and communities as these can be helpful for their well-being and recovery. Maintaining these relationships is also important to unpaid carers (family or friends), but they can be strained by carers’ instigation of, or compliance with, the involuntary detention. Section 17 of the Mental Health Act (1983) in England and Wales allows for temporary leave from hospital, from an hour in the hospital grounds to going home for a few days. However, carers are not always involved in decisions around statutory s.17 leave, even where they are expected to support someone at home. This study aimed to explore how practice can be improved to better involve and support carers around s.17 leave.

**Methods:**

Semi-structured interviews and focus groups were held with 14 unpaid carers and 19 mental health practitioners, including four Responsible Clinicians, in three sites in England in 2021. The research explored views on what works well for carers around s.17 leave, what could be improved and the barriers to such improvements. Transcripts were analysed using reflexive thematic analysis.

**Results:**

Three themes were identified in the analysis: the need for carer support and the challenges surrounding provision; challenges with communication, planning and feedback around s.17 leave; and inconsistency in involving carers around s.17 leave. Permeating all themes was a lack of resources presenting as under-staffing, high demands on existing staff, and lack of time and capacity to work and communicate with carers.

**Conclusion:**

Implications include the need for more funding for mental health services for both prevention and treatment; staff training to increase confidence with carers; and standardised guidance for practitioners on working with carers around s.17 leave to help ensure consistency in practice. The study concluded with the production of a ‘S.17 Standard’, a guidance document based on the research findings consisting of 10 steps for practitioners to follow to support the greater involvement and support of carers.

## Background

In the UK, an estimated 1.5m friends and family members (‘unpaid carers’, hereto ‘carers’) provide substantial support to people experiencing mental health problems [1]. Where an individual’s mental health problems may require them to be detained in hospital for assessment and/or treatment, it is important that the individual maintains contact with their family, friends and communities as this supports their recovery [2]. Maintaining these relationships is also important to carers [3]. Benefits of supporting carers and involving them in patients’ treatment include improvements in the health and well-being of carers and patients’ and carers’ increased satisfaction with services [4, 5]. However, systematic reviews have found that carers tend not to be supported or involved in patients’ treatment [6–8]. In part this may be due to staff lacking confidence in working with carers [9, 10]; concerns around breaching patient confidentiality [9]; or a ward/hospital culture that does not support the involvement of carers [9]. Where inpatient wards do support and involve carers, this can vary between wards and over time [11].

Research has shown that caring can have a significant negative impact on carers’ physical health, employment, relationships, emotional and mental wellbeing, finances and quality of life [7, 12]. Some carers report mental health problems, most notably depression and emotional stress, as a result of caring [13]. The needs of carers are perhaps least likely to be considered during mental health hospital admissions, despite the fact that an admission is likely to have been preceded by a significant deterioration in the mental health of the patient, a corresponding increase in the level of care and emotional distress of the carer, and potentially the trauma of police involvement or involuntary detention under mental health legislation [6], all of which can add to the feelings of guilt and failure experienced by many carers [3, 7].

Carers can be impacted by their involvement in the coercion experienced by the patient who has been involuntarily detained in hospital. Such coercion can take a variety of forms: prior to admission with the intention of keeping the cared-for person safe (administering medication, taking control of finances, confinement in the home, threatening to contact the police or mental health professionals/hospital); leading up to and during the admission if carers request or are ‘complicit in’ legal compulsion (involuntary detention); and following discharge if carers monitor or assist with adherence to community treatment orders in an attempt to prevent hospital readmission [14]. Carer involvement in such coercion can strain family relationships, lead to conflict and impact on trust, resulting in feelings of abandonment for patients and guilt for carers who lack support and are worried about poor care and treatment of the patient in hospital, potentially impacting on carers’ mental and physical well-being [7, 15]. Patients may refuse to see the carer and refuse permission for inpatient staff to discuss their care and treatment with carers [7] which adds to the challenge of inpatient staff supporting and including carers in, for example, planning section 17 leave. In England and Wales, section 17 (s.17) of the Mental Health Act (MHA) 1983 allows the Responsible Clinician (RC) to grant a leave of absence from hospital for those detained. This could include an hour in the hospital grounds, visits to local shops, or going home for a few hours or days. This may be escorted so that the patient is accompanied by a carer and/or member of hospital staff, to mitigate risk, ensure medication adherence, and/or to assess how the patient manages in their home environment to help assess their readiness for discharge. Good practice dictates that planning for discharge should begin at the start of the admission and involve carers [16]. However, research suggests that carers do not feel involved in decision-making, despite being expected to provide support on discharge [7, 17].

Consideration of carers’ experiences and support for policies that involve and support carers in mental health services is international [18, 19]. The United Kingdom has some of the most comprehensive policies for including carers in service user care [18, 20, 21], including the Triangle of Care which aims to develop a therapeutic alliance between service users, carers and mental health practitioners during inpatient stays [22]. However, none address the specific issues facing carers supporting people during s.17 leave.

A recent small-scale study [3] found that carers of people detained under the MHA struggled with anxiety in the lead up to s.17 leave; low mood following leave; stigma from family members or communities who associated detention with criminality; guilt around frequency of visiting the patient; and self-sacrifice in prioritising the patient’s needs over their own. Carers stated that their relationships with practitioners were key to their overall experiences (both good and bad). Notably, carers in that study received very little support, which may have exacerbated the difficulties they faced.

The current study aimed to explore how carers wanted to be involved and supported around s.17 leave and what mental health practitioners felt was feasible in practice, with the aim of developing guidance for inpatient staff in working with carers around s.17 leave.

## Methods

### Study design

A qualitative methodology enabled in-depth exploration of the views of carers, practitioners and RCs. Semi-structured topic guides offered a series of core questions with scope for participants to raise further issues which they felt to be important [23]. Carers, practitioners and RCs were offered individual interviews to ensure that they felt comfortable to speak openly; practitioners also had the option of taking part in a focus group to enable exploration of shared or divergent experiences [23]. Data were collected by telephone or online video call in spring-summer 2021 when Covid-19 restrictions in England meant it was not possible to collect data in-person. Although telephone interviews tend to be shorter than face-to-face interviews and omit the observance of non-verbal cues which can change how something is perceived, evidence suggests they still produce rich descriptive data [24] and indeed can make participants feel more comfortable in sharing accounts of sensitive experiences [25].

The study was conducted across three sites in England (two NHS Mental Health Trusts and a private hospital), to ensure a diversity of experiences of shorter and longer inpatient stays and urban and rural settings.

### Procedures

Eligible carers were unpaid friends or family members of a person with mental health problems who had provided care during a period of s.17 leave within the previous year; with both carer and patient over eighteen years of age. The consent of the patient was not required as the research focused on the views and experiences of the carer. Carers were identified via the research nurse/team in each site through screening records and, in some sites, sharing study information with hospital-based carers’ groups and inviting them to volunteer to take part.

Eligible practitioners worked in inpatient mental health wards or community teams and had experience of working with service users and/or carers during periods of s.17 leave. RCs were eligible if they worked on inpatient mental health wards and had experience of prescribing s.17 leave. Practitioners and RCs were identified by the research nurse/team in each site.

Identified carers, practitioners and RCs were given study information sheets and consent forms with instructions to contact the University research team directly for more information and to ask questions if they were interested. Details of practitioner workshops were also advertised within sites. All participants gave informed consent.

Building upon findings from the earlier study [3], interview questions focused on communication between staff and carers; carers’ understanding of s.17 leave; carer involvement in planning s.17 leave; feedback following leave; support for carers; perceived barriers to enhancing involvement of carers; and potential ways of tackling some of those barriers which were feasible in practice.

Carer participants were each sent a £20 shopping voucher as a ‘thank you’ for their time.

### Ethical considerations

The study raised potential concerns around coercion, anonymity and confidentiality. However, the information sheets stated that participation was voluntary and would be kept confidential. Sites were not informed which carers or staff had taken part in the study. Transcripts were anonymised prior to data analysis and each participant given an ID code. Participants were informed that confidentiality would only be broken if there was a disclosure of harm or unsafe practice. As the questions could potentially be distressing, the information sheet set out the types of questions that would be asked, informed participants that they could take a break or terminate the interview at any time, and provided contact details of support organisations that could offer information, advice or support. The research was approved by an NHS Research Ethics Committee (Ref: 21/NE/0009).

### Data analysis

Demographic data was reported in aggregate by participant type to maintain anonymity. Audio recordings of interviews and focus groups were transcribed verbatim by a professional transcription company, then anonymised by the research team. Transcripts were read and re-read until the researchers were immersed in the data and analysed inductively to generate themes from codes using a reflexive thematic analysis approach [26]. Transcripts were coded in Microsoft Word using the comment function to add codenames and notes. The coding frame was initially devised from the topic guide, then refined iteratively following the re-reading of transcripts and each round of coding. A sample of transcripts were coded by the second author, discrepancies were discussed and the coding frame refined. The potential for conscious or unconscious bias in the design and framing of the questions and/or interpretation and communication of responses [27], was addressed as far as possible through discussion and challenge of the questions, the coding and the analysis among the research team. Themes were identified from the data and written up.

The ‘S.17 Standard’ was produced iteratively through analysis of transcripts and incorporation of suggestions into the topic guides for both carers and practitioners and discussed with each. This could not be a wish list for carers but had to be based on what practitioners also felt would be feasible in practice. The aim was to produce practice guidance by the end of the study, should there be sufficient areas that carers and practitioners agreed upon, and for this to then be evaluated in a future study. The S.17 Standard was also informed through discussion with the project advisory group which consisted of mental health carers, service users and practitioners with experience of s.17 leave.

## Results

### Sample

Thirty-three participants from across the three study sites were interviewed: 14 carers, 15 practitioners and 4 Responsible Clinicians. All carers were interviewed by telephone; practitioners took part in an online focus group (3 focus groups, *n* = 11 practitioners) or individual telephone interview (*n* = 3) or video call (*n* = 1); and RCs were interviewed by telephone (*n* = 1) or video call (*n* = 3). Interviews and focus groups lasted 30–70 minutes.

Carer participants were aged 40 years or older; more than half were female; and all defined as white British. Years in the caring role ranged from less than one year to over 16 years. Carers were the partner or parent of the patient. Practitioner participants, including RCs, were aged 25–69 years; mostly female (*n* = 15); and White British (*n* = 15). Almost half were nurses (*n* = 9). The vast majority had experience of working on inpatient wards (*n* = 17), while two-thirds also had experience of working in the community (*n* = 13). Experience of working in mental health services ranged from 0 to 5 to 16+ years (see Table 1). Four practitioners, at least one from each site, were RCs with 1–13 years’ experience in the role.

**Table 1:** Demographics of the carer and practitioner interviewees

Category	Carers (*n* = 14)	Practitioners (*n* = 19)
**Age**		
18–24	0	0
25–39	0	10
40–54	5	5
55–69	7	1
70+	2	0
Not provided	0	3
**Gender**		
Female	8	15
Male	6	4
**Ethnicity**		
White British	14	15
Black British	0	1
Asian	0	1
Not provided	0	2
**Years in caring role (carers) / working in mental health services (practitioners)**		
0–5	6	4
6–10	3	4
11–15	3	4
16+	2	4
Not provided	0	3
**Has inpatient experience**	-	17
**Has community experience**	-	13
**Relationship to patient**		
Partner	6	-
Parent	8	-
**Professional background**		
Nurse	-	9
Social worker or Occupational Therapist	-	3
Psychologist	-	2
Healthcare assistant	-	2
Psychiatrist	-	2
Not provided	-	1

### Themes

Three key themes emerged from the data: the need for carer support and the challenges surrounding provision; challenges with communication, planning and feedback around s.17 leave;; and inconsistency in involving carers around s.17 leave.

### 1. The need for carer support and the challenges surrounding provision

Carers spoke of the trauma they experienced in the build up to and during the crisis which preceded the hospital admission. One carer explained:

*“There’s the shock of somebody going into a psychotic episode… nothing can prepare you for that at all…it knocks you back, to be honest.” (Carer 8)*.

Some carers identified a need to emotionally recharge following the admission and mentally prepare for the first period of s.17 leave. The emotional stress on the carer could be compounded if leave was granted quickly, before the carer had had time to recuperate, and if they thus felt unable to support the leave which could then cause feelings of guilt. Carers wanted to be involved in decision-making around s.17 leave for multiple reasons, including to ensure that leave was planned for when they felt emotionally prepared to support it.

### The necessity of support for carers

Carers spoke of the toll on their own mental health when they felt unsupported by services following a traumatic incident around the leave:

*“I’m sure that I have been more affected by what happened because I didn’t at the time get a chance to talk about how I felt at the time, with staff… if there’s a traumatic, if something’s gone wrong, there definitely needs to be a lot more support for the poor carer.” (Carer 10)*.

Most carers felt that they would benefit from help, advice and support. One carer made a comparison to the support offered to staff and argued that carers also require support:

*“Staff get clinical supervision… we should have something.” (Carer 12)*.

The carer described how, over time, they had come to realise that support for carers was essential for the carers, the patients and for the system to keep functioning:

*“If the stress is too much for families, what’s going to happen when that person is well enough to go back to the community and their system of support is not there anymore because it’s broken, because nobody provided them with any support.” (Carer 12)*.

However, several carers acknowledged that they would not feel comfortable or confident in asking for help and would only access support if it was offered:

*“Am I failing because I’m having to ask for support or ask for help? You know, we’ve all got that in us I think, that pride of I want to do this myself… and it isn’t always the best way by a long way. And it is that bit easier if it’s put to you, ‘would this be useful to you?’” (Carer 8)*.

### Types of support offered and desired

A minority of carers reported that they had been offered some support, and experiences of this were mixed. A carer who had had sessions with a psychologist reportedly found this helpful but felt too few sessions were offered; one carer found a carers support group to be quite helpful while others had not wanted to get involved as they felt too raw at that point in time; and the offer of a voucher for a massage was reported to be ‘nice’ but unhelpful.

Some carers reported wanting to talk through what had happened in the build up to the admission and seek assurance or advice on their own response to the situation and to the patient. Others stated they wanted more specific help, notably practical support and/or somebody to listen:

*“When we’re talking about support, we don’t want monetary support… [We want] practical support… support like having an advocacy service.” (Carer 12)*.

### Recognising carers

Most carers noted they had not been offered any support in their role as a carer. Some accepted this as a function of stretched resources and practitioners’ focus on the patient. This was echoed by practitioners across different sites who noted *“the role of a staff nurse in an inpatient setting just doesn’t stretch to that [carer support] unfortunately.” (Practitioner 4)*.

There was also an underlying sense that the culture on some wards did not recognise or value carers suggesting they were not seen as a significant part of the patient’s recovery:

*“Our priority is always, and I know it sounds awful, it’s always the patient, and not the visitor. That’s where we prioritise.” (Practitioner 13)*.

Indeed, the description of carers as ‘visitors’ suggests a lack of recognition of carers as an integral part of the team caring for the patient.

An RC suggested staff training could be helpful, particularly for those who hadn’t worked with carers and/or who didn’t see the value of having carers as part of the team.

An additional challenge was that carers did not always self-identify as carers, instead seeing caring for their family member as their duty, and thus conversations about carer support did not happen.

Some practitioners identified that the duration of the inpatient stay may not be long enough for carer support to be put in place, particularly on acute wards, with a suggestion that carers’ support could instead be the responsibility of community mental health teams, with just an initial discussion around carer support from ward staff.

A small minority of carers were clear that they did not want carer support. For some this was explained as wanting resources to be directed at helping the patient, which in turn would help them. This aligned with some carers not identifying as such and not accepting that they could benefit from support at this challenging time. Other carers stated that they preferred to rely on their own personal support networks rather than have to repeat their story to numerous professionals which suggests an inconsistency of practitioners and a challenge with joined-up working between services. This suggests that carer support is not one-size-fits-all, but rather needs to be person-centred, tailored to the needs and wishes of the individual carer and that services could be more carer-focused and joined-up to minimise repetition of often traumatising experiences for carers.

### 2. Challenges with communication, planning and feedback around s.17 leave

Many carers reported that communication around s.17 leave was either absent or unclear with some not knowing what s.17 leave was. This lack of information sometimes spanned numerous admissions over many years and potentially across different wards and hospitals:

*“We had no idea, nobody ever told us, that he could come home on leave from hospital… I presume that [hospital] must have assumed that we knew [what s.17 leave was] seeing as [patient had] spent six years somewhere else, but it was a case of we didn’t know, and we didn’t know we didn’t know.” (Carer 11)*.

Most carers reported being unaware of any ‘rules’ or expectations around leave or what to do if there was a concern during the leave:

*“You want to know something about what the expectations are of you… things like if she gets up and goes to the toilet, are you supposed to go with her or can she go on her own… what is she allowed to do and what is she not. And what are we supposed to be doing to make sure she does the things she’s supposed to do… [Also, following a serious incident during the leave] nobody had told us what we should do in that situation. There had been no discussion about whether we should take her to A&E or whether we should phone [the ward] and stuff like that…” (Carer 12)*.

Carers suggested that a chat with staff prior to leave about how the patient was getting on would be helpful, as would a written information sheet about what to do if there was an incident during leave.

### Planning s.17 leave

Only two of the 14 carers interviewed gave positive examples of being closely involved with planning s.17 leave. In each case the carer was told that leave was available and was then able to choose or suggest times that would be convenient for themselves:

*“It was more me giving them notice, than the other way around.” (Carer 4)*.

In both cases the carer was male, caring for their wife. However, other male carers did not report the same sense of control. Planning the leave appeared less stressful for the carers who requested leave, which in turn may reflect the carers’ readiness to support the patient at home.

Many carers perceived that the notice given before leave was inadequate. For example, some carers were asked if they could support s.17 leave the same day, which was particularly challenging for those with work, childcare or other commitments, who reported feeling angry or frustrated at being expected to drop/change plans at short notice. For others, there was a need for reassurance ahead of agreeing to any leave:

*“It’s not acceptable just to ring up in the morning or an hour before… if you’re working in a hospital setting, then you’re dealing with the person once they arrive. I’ve been dealing with the person forever and [there’s a] level of reassurance I need about [how]… it’s going to be manageable for me at home.” (Carer 7)*.

Some practitioners noted that lack of time and capacity meant they were not often able to discuss the decision to grant leave and the patient’s recovery with the carer which fuelled carers’ apprehension. Most practitioners acknowledged that carers were often asked to support s.17 leave at short notice and explained that this was often due to a lack of time and staff resource.

RCs argued that the planning of the s.17 leave usually did include carers, particularly those who lived with the patient. One RC reported asking ward staff to contact carers to find out how much leave they were willing to support before this was written up. Where this did not happen, a number of reasons were cited. These were primarily issues within the patient-carer relationship suggesting that the home environment could cause a deterioration of the patient’s mental health or was not conducive to the patient’s recovery, for example suspicions or a history of abuse, neglect or exploitation, mental health problems among other family members, requests from patients not to contact carers, or where there was experience of carers being unreliable and not turning up for leave or refusing to return the patient to hospital following leave.

Some practitioners noted that restrictions around Covid-19, in particular the inability for carers to visit the ward, had reduced opportunities for carers to see patients in the ward environment and for staff to update carers on patient progress.

A further issue around planning s.17 leave was that some carers did not know where to go or what to do during the leave, particularly if the hospital was not close to home and the area was unfamiliar:

*“And advice about the local area because if you don’t live in the area you don’t know where to go… and that just on top of the emotional strain and the physical effort of going, and all the rest of it, it just adds one more layer of difficulty that’s unnecessary.” (Carer 11)*.

A list of suggestions about where to go on the leave was requested by a number of carers and reported to be a good idea by most practitioners.

Carers gave examples of s.17 leave arrangements being changed by ward staff at short notice and not communicated to, or agreed with, the carers. Examples included changes to the length of the leave, whether leave was escorted by a member of staff, and any restrictions on where the patient may go. Such changes had caused considerable distress to the carers.

### Feedback following leave

Practitioners appeared to view carer feedback as intelligence about the patient’s recovery and abilities in the community. Carers too seemed to view feedback as providing information to assist practitioners with the care of the patient. Neither practitioners nor carers acknowledged feedback as an opportunity for the carer to debrief or have their own experience recognised unless there had been an incident during leave.

For example, one RC asserted that feedback following s.17 leave was essential to help them understand where the patient was in their recovery journey and to inform their care:

*“You are trying to get them back into their home environment, so getting that feedback is crucial really.… it makes it a lot easier to understand what level of recovery somebody is at.” (RC 2)*.

A few practitioners commented that carer feedback was only necessary if something noteworthy had occurred during the leave. Otherwise, some practitioners felt that they were ‘overstepping’ (*Practitioner 4*), intruding on carers’ time and/or wasting their own time. However, the majority reported that feedback should be sought following each episode of s.17 leave.

Almost all practitioners and RCs acknowledged that - where staffing and the absence of critical incidents on the ward permitted - staff should ask carers for feedback in person immediately following the leave or, if this was not possible, phone carers as soon as possible thereafter. Most carers and practitioners agreed that the onus should be on staff to ask carers for feedback. For example, one carer explained that some carers may lack confidence or have had negative experiences with mental health services and not feel comfortable making contact. Practitioners lamented lacking the time, primarily due to staff shortages, to always ask carers for feedback following leave.

Most participants also agreed that carers should be asked to provide feedback in private, without the patient present, to protect the carer-patient relationship and enable carers to give feedback openly.

Some practitioners expressed lacking in confidence in speaking with carers and/or perceived this lack of confidence in colleagues. Different reasons for this unease were suggested: concern/fear over saying the wrong thing; uncertainty around patient confidentiality; limited knowledge around s.17 leave, especially for newer/unqualified staff; lacking time to speak with carers, especially due to staff sickness and vacancies; and staff struggling with having difficult conversations with carers. Practitioners suggested that communication challenges might be improved through specific training around s.17 leave for all staff; having a designated worker with time allocated to speak with carers; and filling vacant positions.

### 3. Inconsistency in involving carers around s.17 leave 

The third theme highlighted the extent of variation in practice among practitioners, RCs and wards owing to different ward cultures or leadership styles. Differences included information given to carers, whether or not carers were invited to ward rounds, whether staff felt comfortable engaging with carers, and whether consultants actively encouraged/sought feedback from carers. It was notable that such variation existed both within and between organisations and again seemed to be related to the ward culture and the particular RC.

To encourage consistency and closer working with carers, some practitioners advocated having staff dedicated to working with carers, whether for example a named nurse on the ward or *‘a middle man, an advocate for carers’ (Practitioner 4)* either on or outside the ward.

Carers, practitioners and RCs suggested that a consistent, standardised approach to co-working with carers had the potential to benefit patients, carers and practitioners alike. For example, carer participants with experience of multiple detentions noted that it would be helpful if information and procedures were standardised across wards and hospitals so that carers could expect a level of involvement and support if and wherever the person they cared for was detained in hospital. Numerous practitioners agreed, calling for clear policies and procedures that were effectively put into practice.

### Development of the ‘S.17 Standard’

**Fig. 1:**
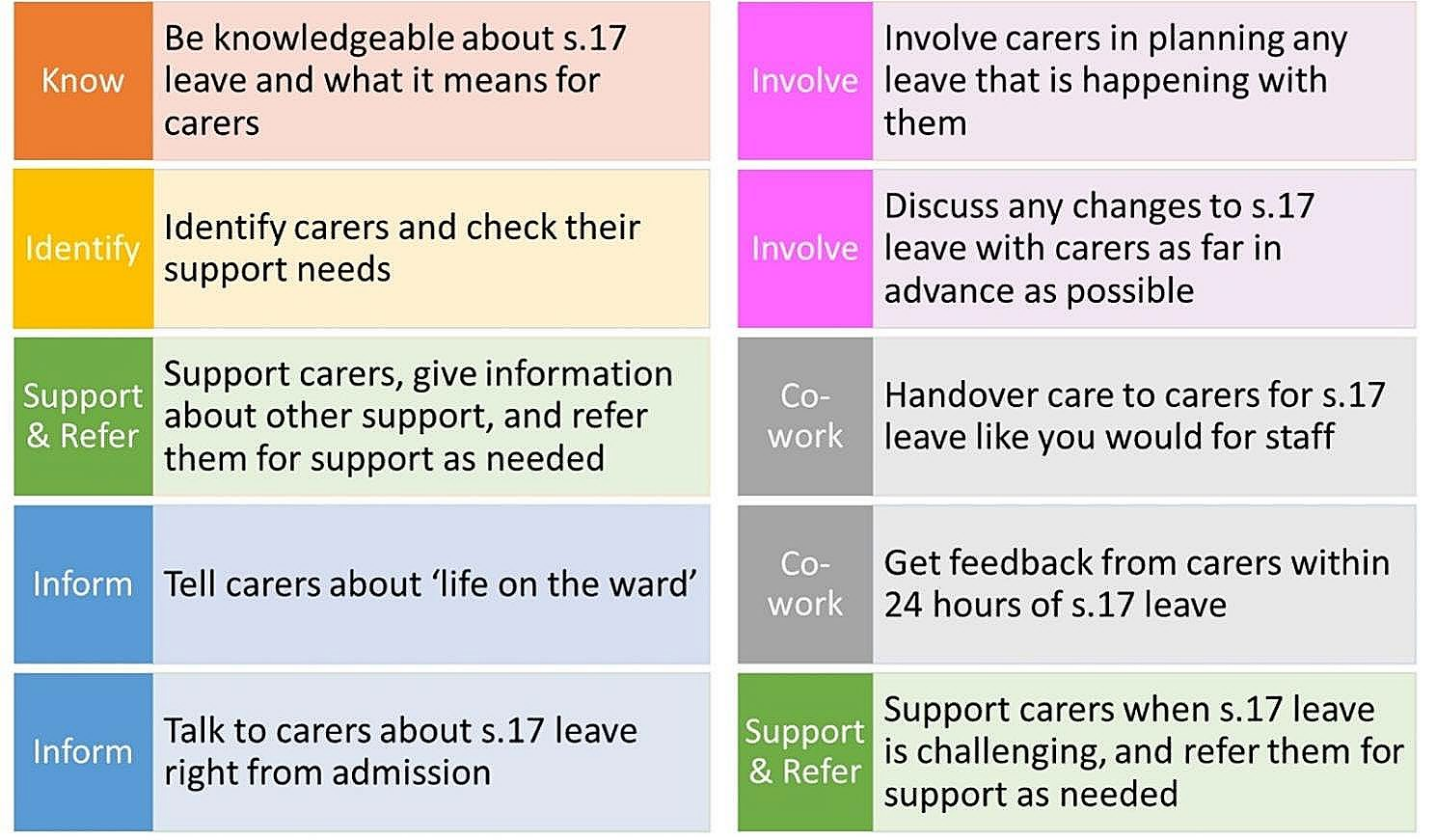
The s.17 Standard for Carers

At the conclusion of the study, the research team produced a ‘S.17 Standard’, a guidance document developed out of the research findings and refined by the research team and the project advisory group. The S.17 Standard consists of 10 steps for practitioners to follow to increase and encourage the greater involvement and support of carers around s.17 leave. The Standard covers the need for staff training to improve understanding of the importance of working with carers in general and specifically around s.17 leave, identifying and responding to carer support needs, discussing s.17 leave with carers and answering any questions they may have, involving carers in planning s.17 leave, agreeing any changes to leave with carers ahead of the leave, seeking feedback from carers following leave, and supporting carers following a difficult leave experience (Fig. 1).

The Standard thus aimed to offer the support and involvement that carers said they wanted and that practitioners felt was feasible in practice. The Standard will be fully articulated in a practice guidance manual to assist practitioners to embed it in their practice.

## Discussion

The research identified three key themes: carers’ need for support and issues surrounding the provision of support; communication challenges between staff and carers, notably around planning s.17 leave and feedback; and the lack of consistency in the involvement of carers around s.17 leave. Permeating all themes was a lack of resources. Whilst most practitioners stated a desire to work with carers around s.17 leave, high demand and high levels of acuity on inpatient wards coupled with staff shortages and under-funding resulted in a lack of time and capacity for many practitioners to involve, support and communicate with carers as much as they would like, echoing findings from other studies [19, 28]. A resounding implication is thus the need for more funding for mental health services, both for mental health hospitals/units to facilitate holistic involvement and support for carers, but also in the community to minimise the numbers of people requiring detention in hospital under the Mental Health Act. The need to tackle structural inequalities and the social determinants of poor mental health underpins much of the debate. However, the need for increased resourcing and addressing inequalities is not the only implication.

The study highlighted carers’ need for support with respect to their own trauma around the build up to the hospital admission, the admission itself and any incidents during the s.17 leave. This could include carers’ own mixed feelings of their involvement in the involuntary detention and any resulting impact on their relationship with the patient. Carers said they valued or would have appreciated emotional and/or practical support. This resonates with findings from an Australian study which found that carers who were referred for support found this helpful [29]. Other studies have identified carer-reported benefits of psychoeducation groups for carers [30], including those for carers of inpatients [31]. Such carer support should be available to carers independently of the patient, in line with NICE guidelines [21]. The study also highlighted challenges with the existing system and identified why some carers may not access carer support, including not identifying as a carer, not wanting to take up resources that could be directed towards the patient, and not wanting to repeat their emotionally delicate story to numerous professionals. This suggests that carer support needs to be person-centred and also cognisant of mental health contexts [32], including, for example, some carers' feelings of guilt and trauma around their role in the involuntary detention [14, 15].

Many carers reported feeling invisible when attempting to communicate with inpatient ward staff, as noted elsewhere [7, 9, 33]. Some practitioners acknowledged this, with explanations that they or their colleagues lacked confidence in speaking with carers over fears of saying too much, saying the wrong thing, or not having sufficient information about the patient to know what to say to the carer. This finding resonates with other studies which found that some staff struggle to engage with carers [9, 10, 19] and/or have limited confidence in working with carers [19], potentially due to concerns around patient confidentiality [9, 33]. Some practitioners felt that the consultant and/or ward culture did not overtly support or recognise the role of carers and this impacted their own practice, supporting findings from other studies [5, 9, 33].

A lack of consistency was also noted by carers and some practitioners regarding the information shared with carers, carer involvement in planning s.17 leave, requests for carer feedback following leave, and support offered to carers throughout the admission and following a traumatic incident during leave. S.17 leave is a mechanism of the Mental Health Act and as such flexibility around decision-making may be particularly complex. As inpatient treatment is provided under statutory intervention, inpatient staff may feel less compelled to involve carers. However, variation within and across study sites again potentially reflected ward culture, with some RCs and wards more carer aware and inclusive of carers than others, supporting the involvement of carers in deciding when s.17 leave may happen, for example, suggesting that carer involvement is possible even in the context of involuntary detention.

Carers and practitioners reported challenges around communication and carers struggled with a lack of knowledge and information about many aspects of s.17 leave. Discussions between practitioners and carers about s.17 leave, and perhaps the provision of written information, is likely to increase carer knowledge and confidence around s.17 leave and ensure that carers have easy access to key information, which support findings about communication from other studies [7, 9]. Staff training around the importance of working with carers and ‘having difficult conversations’ could also help increase staff confidence and improve staff-carer communication and co-working. Whilst this may be hampered by issues around patient confidentiality, in particular where the relationship between carer and patient is strained perhaps linked to the actual or perceived involvement of carers in the compulsory detention, generic information about what happens on the ward could still be shared with carers.

Standardised guidance for practitioners on working with carers around s.17 leave could help to ensure consistency in practice across inpatient wards and hospitals. This could include good practice around planning s.17 leave with carers to ensure carers’ needs and other commitments are also accounted for; and practitioners seeking feedback from carers following leave. Such guidance could include staff training around the importance of communicating with and including carers. Studies have shown that such training improves staff confidence and is welcomed by staff [34], whilst carers value improved communication, information sharing and emotional support [8, 34]. This also resonates with best practice guidance from the UK Triangle of Care which highlights the importance of training staff in carer awareness and providing/referring carers to support services [22].

The development of the ‘S.17 Standard’ aims to provide such national guidance for practitioners, to offer a standard approach to involving and supporting carers around s.17 leave, though it needs to be tested in diverse practice settings to assess how it works in practice, limitations, and the feasibility of implementing such guidance in busy hospital wards. The guidance will be accompanied by a practice guidance manual setting out the ten steps, practical ways of achieving them and the rationale behind each step to aid understanding and thus compliance; a summary practice guidance document; a leaflet for carers explaining s.17 leave as this was a key area for improvement; posters and ‘business cards’ to remind staff about the S.17 Standard; and training videos for practitioners created by the research team consisting of a series of short films covering different sections of the guidance (for example, what to do before leave, during leave, and following leave).

### Limitations

Use of gatekeepers to approach prospective participants and self-selection in recruitment would have limited the representativeness of participants. However, despite being a small qualitative study, data saturation was achieved as no new themes emerged from the later interviews/workshops [35]. A key limitation is the lack of diversity among carer participants, with all self-identifying as White British and none as carers of parents with mental health problems. The study sites covered a mix of ethnically diverse areas; however, the study information pack was only available in English which may have excluded some carers. It would be useful to conduct further research with carers from diverse ethnic backgrounds and with carers who have more diverse relationships to the patient including adult carers of parents with mental health problems and also young carers.

## Conclusions

This study found the challenge of resourcing, notably staff shortages, impacted on staff time to communicate with carers about s.17 leave, including planning s.17 leave, obtaining carer feedback following leave, and carer support. However, not all challenges were resource-dependent. Some carers felt staff did not value them, while some practitioners acknowledged a lack of confidence in speaking with carers. Steps to improve carer support and involvement around s.17 leave could thus include training for staff about the benefits of working with, and having difficult conversations with, carers; information for carers about s.17 leave; and clear national guidance setting out a consistent approach for practitioners to work with carers around s.17 leave. These steps would need to be cognisant of the additional complexity of the context including the emotional distress of carers who may have been ‘complicit’ in the involuntary hospital detention with the subsequent strains on family relationships and inpatient staff unsure whether working under the Mental Health Act may limit flexibility around carer involvement. Such steps could improve staff-carer relationships, carer wellbeing, and potentially carer-patient relations, to the benefit of all. The study concluded with the production of a ‘S.17 Standard’ incorporating all of the above in to 10 steps for inpatient ward staff to follow to ensure the involvement and support of carers around s.17 leave. The next step is for the Standard to be tested in practice to look at indicative outcomes for carers and implementation issues in busy hospital wards before potentially being adopted as national guidance around s.17 leave.

## Data Availability

The datasets generated and analysed during the current study are not publicly available due to the potential for individual privacy to be compromised but are available from the corresponding author on reasonable request.

## Abbreviations

RC: Responsible Clinician usually the consultant in charge of patient care
MHA: Mental Health Act (1983) in England and Wales
s.17 or s.17 leave: Sect. 17 of the Mental Health Act (1983) through which Responsible Clinicians can grant temporary leave from the hospital for patients who are detained under the Act
